# Supraphysiological testosterone levels from anabolic steroid use and reduced sensitivity to negative facial expressions in men

**DOI:** 10.1007/s00213-023-06497-2

**Published:** 2023-11-22

**Authors:** Morgan Scarth, Lisa Evju Hauger, Per Medbøe Thorsby, Siri Leknes, Ingunn R Hullstein, Lars T. Westlye, Astrid Bjørnebekk

**Affiliations:** 1 Anabolic Androgenic Steroid Research Group, Section for Clinical Addiction Research, Division of Mental Health and Addiction, Oslo University Hospital, Postbox 4959, Nydalen, 0424 Oslo, Norway; 2 Department of Psychology, University of Oslo, Oslo, Norway; 3 Hormone laboratory, Department of Medical Biochemistry and Biochemical endocrinology and metabolism research group, Oslo University Hospital, Oslo, Norway; 4 Institute of Clinical Medicine and University of Oslo, Oslo, Norway; 5 Department of Diagnostic Physics, Oslo University Hospital, Oslo, Norway; 6 Norwegian Doping Control Laboratory, Oslo University Hospital, Oslo, Norway; 7 NORMENT, Division of Mental Health and Addiction, Oslo University Hospital & Institute of Clinical Medicine, University of Oslo, Oslo, Norway; 8 KG Jebsen Centre for Neurodevelopmental Disorders, University of Oslo, Oslo, Norway

**Keywords:** Anabolic-androgenic steroids, Testosterone, Social cognition, Emotion recognition, Dependence

## Abstract

**Rationale:**

Anabolic-androgenic steroids (AAS) are used to improve physical performance and appearance, but have been associated with deficits in social cognitive functioning. Approximately 30% of people who use AAS develop a dependence, increasing the risk for undesired effects.

**Objectives:**

To assess the relationship between AAS use (current/previous), AAS dependence, and the ability to recognize emotional facial expressions, and investigate the potential mediating role of hormone levels.

**Methods:**

In total 156 male weightlifters, including those with current (*n* = 45) or previous (*n* = 34) AAS use and never-using controls (*n* = 77), completed a facial Emotion Recognition Task (ERT). Participants were presented with faces expressing one out of six emotions (sadness, happiness, fear, anger, disgust, and surprise) and were instructed to indicate which of the six emotions each face displayed. ERT accuracy and response time were recorded and evaluated for association with AAS use status, AAS dependence, and serum reproductive hormone levels. Mediation models were used to evaluate the mediating role of androgens in the relationship between AAS use and ERT performance.

**Results:**

Compared to never-using controls, men currently using AAS exhibited lower recognition accuracy for facial emotional expressions, particularly anger (*Cohen’s d* = −0.57, *p*_*FDR*_ = 0.03) and disgust (*d* = −0.51, *p*_*FDR*_ = 0.05). Those with AAS dependence (*n* = 47) demonstrated worse recognition of fear relative to men without dependence (*d* = 0.58, *p* = 0.03). Recognition of disgust was negatively correlated with serum free testosterone index (FTI); however, FTI did not significantly mediate the association between AAS use and recognition of disgust.

**Conclusions:**

Our findings demonstrate impaired facial emotion recognition among men currently using AAS compared to controls. While further studies are needed to investigate potential mechanisms, our analysis did not support a simple mediation effect of serum FTI.

**Supplementary Information:**

The online version contains supplementary material available at 10.1007/s00213-023-06497-2.

## Introduction

Anabolic-androgenic steroids comprise the male hormone testosterone and synthetic derivatives, typically consumed to increase muscle mass, contributing to their popularity within the weight-lifting and body-building communities. Global lifetime prevalence is estimated to be 3.3% (6.4% among males, 1.6% among females); however, prevalence varies greatly by geographic location and is elevated among certain populations including bodybuilders (24.5%), people with substance use disorders (28.3%), and people in prison (28.5%) (Havnes et al. 2020a; Havnes et al. 2020b; Nakhaee et al. 2013; Sagoe et al. 2014). A number of side effects are associated with high-dose exogenous androgen use, including somatic, psychiatric, and cognitive symptoms (Barbosa Neto et al. 2018; Christoffersen et al. 2019; Christou et al. 2017; Hauger et al. 2021; Hauger et al. 2020; Kanayama et al. 2013; Thiblin et al. 2015; van Amsterdam et al. 2010). Furthermore, approximately one-third of people who use AAS develops a dependence and are often at higher risk for undesired side effects as a result of increased dose and prolonged use (de Zeeuw et al. 2023; Pope Jr. et al. 2014).

AAS use is associated with anxiety, depression, personality disorders, and increased aggression (Chegeni et al. 2021; Hauger et al. 2021; Jørstad et al. 2023; Piacentino et al. 2015). Aggressive and antisocial behaviors have been associated with impaired ability to recognize and process emotional expressions, suggesting that the association between AAS and aggression may be partially due to deficits in social cognitive abilities (Crick and Dodge 1994; Winter et al. 2017). Social cognitive skills include the ability to recognize and infer emotional states of others and are necessary for non-verbal communication (Collin et al. 2013). Perception and interpretation of social information, including facial expressions, contribute to cognitive processes such as empathy, cooperativity, and decision-making. Hormones including testosterone have been implicated in facial emotional processing (Romero-Martínez et al. 2021) which is also supported by the density of androgen receptors in neural structures required for emotion recognition and processing including the prefrontal cortex, amygdala, and hippocampus (Beyenburg et al. 2000; Fusar-Poli et al. 2009; Nuñez et al. 2003; Simerly et al. 1990).

Altered hormone levels resulting from AAS use may partially explain the mood and behavioral effects of these substances (Daly et al. 2003). However, the influence of sex hormones on social cognition is not clear. While women appear to more accurately identify anger and display greater empathy, it is not clear if these sex differences can be attributed to differences in androgen levels (Andric Petrovic et al. 2019; Baron-Cohen et al. 2005; Di Tella et al. 2020). Baseline testosterone appears to be weakly positively correlated with human aggression, with a stronger association in males than females, and no clear causal link has been established (Geniole et al. 2020). The relationship between testosterone and social cognition is likely moderated by age, where higher testosterone in younger men is associated with decreased theory of mind (Grainger et al. 2021). In addition, prior research has demonstrated that salivary testosterone is negatively correlated with emotion recognition for disgust and fear in young, healthy male participants (Rukavina et al. 2018). In certain populations displaying antisocial behaviors, higher levels of testosterone have been associated with poorer perspective taking and emotional recognition (Comes-Fayos et al. 2022; Romero-Martínez et al. 2013).

The influence of exogenous testosterone on social cognition likely depends on sex. While low doses (0.5 mg) of exogenous testosterone appear to reduce empathic behavior and interpersonal trust in women, commonly prescribed doses of testosterone gel (100 mg) did not influence the ability to infer others’ emotional states in healthy young men in a placebo controlled trial (Bos et al. 2010; Nadler et al. 2019; van Honk et al. 2011). However, AAS are often taken in doses exceeding endogenous male testosterone levels by 10–100 times, where high levels of testosterone suppress follicle stimulating hormone (FSH) and luteinizing hormone (LH). This can disrupt natural testosterone production, leading to hypogonadism, characterized by low testosterone and elevated FSH and LH (Brower 2002; Christou et al. 2017; Dandona and Rosenberg 2010; Oduwole et al. 2021). Thus, while exogenous testosterone in doses equivalent to those prescribed for medical reasons may not influence cognitive empathy, previous research from our group indicates that AAS use is associated with deficits in emotional recognition from biological motion and theory of mind (Hauger et al. 2019a; Vaskinn et al. 2020). These social cognitive consequences may be partially explained by endocrine disruption, as people currently using AAS will experience elevated testosterone levels, and those who have permanently or temporarily ceased use often experience low levels of endogenous testosterone. However, the relationship between AAS use and facial emotional recognition has not yet been explored.

AAS dependence is associated with increased challenges in mental and physical health relative to those without dependence, including deficits in social cognitive domains (Hauger et al. 2019a; Vaskinn et al. 2020). This may be partly explained by neurobiological factors influencing magnetic resonance imaging (MRI) based measures of cortical thickness in frontal brain regions and functional brain connectivity, as has been associated with AAS dependence (Bjørnebekk et al. 2017; Hauger et al. 2019b; Westlye et al. 2017). Additionally, AAS dependence likely shares underlying mechanisms and risk factors with other substance use disorders which may contribute to these findings, including personality disorders and executive dysfunctions (Scarth et al. 2022). Previous research demonstrates associations between substance or alcohol use disorder and deficits in facial emotion recognition (Castellano et al. 2015). Furthermore, these deficits may persist over time, despite abstinence (Rupp et al. 2021). While facial emotion recognition accuracy has been evaluated among individuals with psychoactive substance dependence, this has not yet been examined within AAS dependence.

The current study aims to examine the relationships among AAS use, sex hormone levels, and social cognition in a sample of men who currently or previously used AAS, and weight-lifting controls. Additionally, we will investigate differences in facial emotional recognition among men with and without AAS dependence. Based on the literature reviewed above, we hypothesize that men currently using AAS will demonstrate lower accuracy in facial emotion recognition and will significantly differ in hormone levels relative to those who have ceased use and controls. Further, we expect that men with AAS dependence will demonstrate greater deficits than those without AAS dependence. Lastly, we expect that hormone levels, specifically testosterone, will partially mediate the relationship between AAS use and accuracy of facial emotion recognition.

## Methods

### Participants

The study sample is drawn from a longitudinal study of the effects of long-term androgen use on cognition, brain, and cardiovascular health (Bjørnebekk et al. 2021; Bjørnebekk et al. 2017) and consists of 171 adult men involved in heavy strength training. Participants were recruited via social media, online forums, and webpages targeting individuals interested in heavy weight training or bodybuilding. Additionally, posters and flyers were distributed in selected gyms in Oslo, Norway, and recruitment through “snowball sampling.” The participants were men who previously or currently used AAS (*n* = 94), reporting at least one year of cumulative AAS use (summarizing on-cycle periods), or men who had never used AAS or equivalent doping substances, but engaged in heavy resistance training and were able to bench press 100 kg for at least one repetition (“WLC,” *n* = 77). Participants who reported lifetime AAS were further divided into two groups based on current use status; those using AAS at the time of testing (“On”), and those who had ceased use at the time of testing (“Off”). AAS use status, and thus group membership, was confirmed with a combination of urine and blood samples, as well as self-reported time since quitting or time since last use, where those who reported previously using AAS and had a negative urine test, and reported ceasing use for more than 3 months were categorized as *Off*. All participants were 18 years of age or older. The present sample partially overlaps with the sample assessed in previous studies of other aspects of social cognition (Hauger et al. 2019a; Vaskinn et al. 2020), though data was collected at different time points (*n* = 78 from previous sample, *n* = 78 newly recruited).

Urine samples were collected and analyzed for AAS use with gas and liquid chromatography tandem-mass spectrometry at the WADA-accredited Norwegian Doping Laboratory at Oslo University Hospital (Hullstein et al. 2015). The criteria to determine AAS use were (1) urine samples positive for synthetic anabolic androgenic compounds (2) a testosterone to epitestosterone ratio (T/E) > 15, in accordance with previous findings (Bjørnebekk et al. 2021; Bjørnebekk et al. 2017; Hullstein et al. 2015).

Demographic and other clinical data was assessed using an electronic self-report questionnaire. Participants were asked about their AAS use with interviews and questionnaires, including motives behind their usage, age of onset, administration pattern, years of use, length of cycles and number of life-time cycles, side-effects, and average weekly dosage, where in the cycle they were at the time of assessment, and whether and when they had ceased using AAS. IQ was measured using the Vocabulary, Similarities, Matrix Reasoning, and Block Design scales of the Wechsler Abbreviated Scale of Intelligence (WASI), and years of education were self-reported (Wechsler 1999). For further information regarding data collection see Bjørnebekk et al. (2017).

### AAS dependence

Lifetime AAS dependence was evaluated using the Structured Clinical Interview for DSM-IV Axis II Disorders (SCID-II) for substance dependence, adapted for AAS by experts in the field, and has been found to have sufficient reliability and validity (Ip et al. 2012; Kanayama et al. 2009; Pope Jr. et al. 2010). The instrument captures essential characteristics of AAS use, and the degree to which the pattern of use affects the life, physical, and mental health of the user. The interview was administered by trained study personnel and includes seven symptoms of AAS dependence. Interviewers rated the symptoms on a scale from 1 to 3 (absent, subthreshold, present). Participants were categorized as “dependent” if three or more of the criteria were fulfilled at any time while they were using AAS.

### Emotional recognition task

**Fig. 1:**
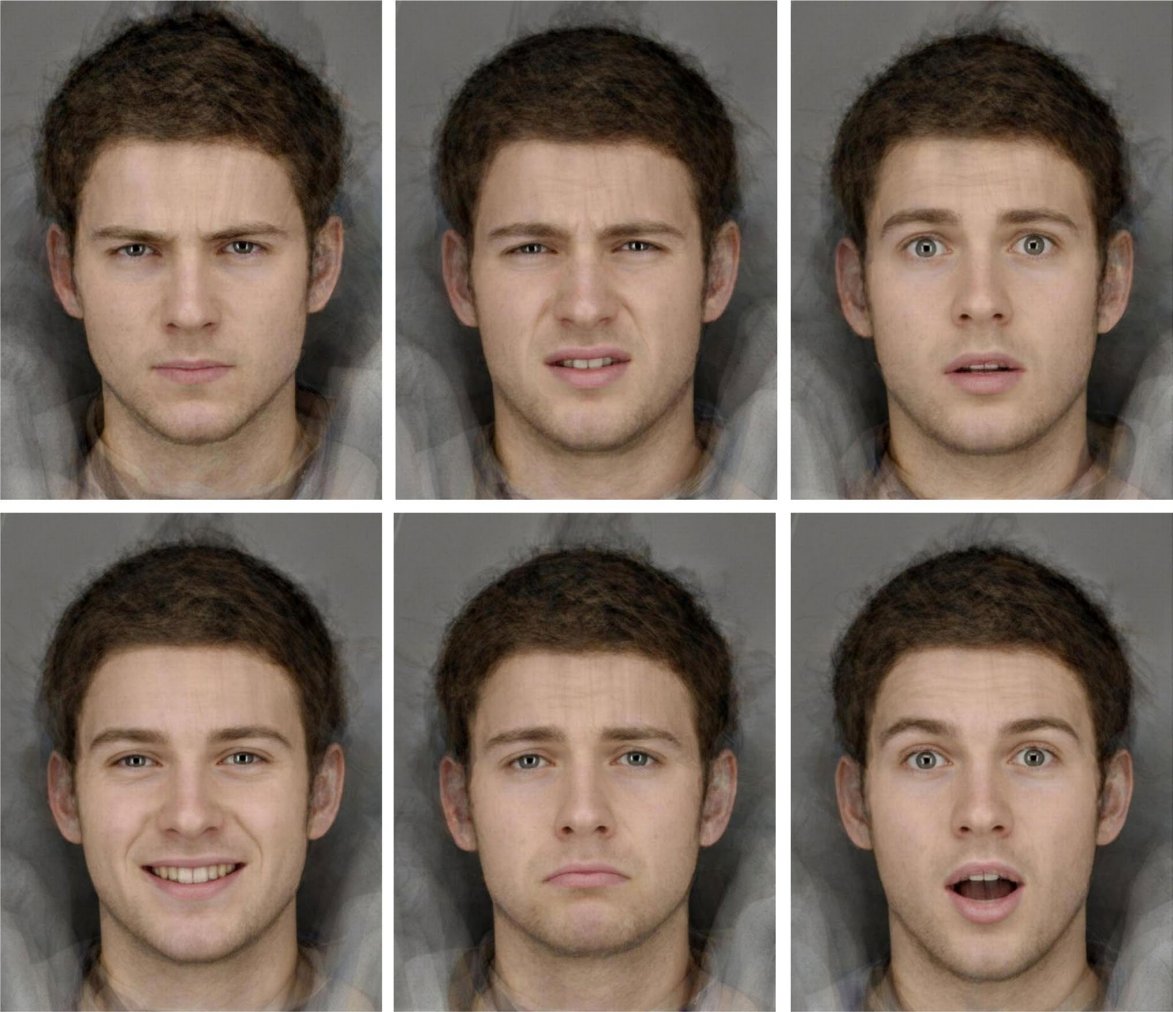
Sample of faces depicting each emotion (clockwise from top left: anger, disgust, fear, happiness, sadness, and surprise) from the CANTAB Emotional Recognition Task.© Copyright 2018 Cambridge Cognition Limited. All rights reserved

The participants completed the emotion recognition task (ERT) from a computerized cognitive battery, the Cambridge Neuropsychological Test Automated Battery (CANTAB) (CANTAB 2016). In this task, the participants viewed computer-morphed images generated from the facial features of real male and female individuals. Each participant was shown eight images of six facial emotions for 200 ms each expressing specific emotions in varying intensities over a total of 48 trials. The specific emotions depicted were sadness, happiness, fear, anger, disgust, and surprise, an example of each emotion is shown in Fig. 1. After each face was shown, the stimulus is covered with a grey rectangle for 250 ms before the participant was presented with six buttons, from which the participant then selected which emotion the face displayed. Participants were informed they should try to select a button as quickly they can, that some faces are more difficult to read than others, and that they may have to guess which emotion was depicted. For each emotion, accuracy (proportion correct responses) and latency (median response time for all responses) were recorded, in addition to overall accuracy and median response time. The test was completed in 6–10 min.

### Hormones

Blood tests were taken at the department of cardiology, Oslo University Hospital, Oslo, Norway. The blood was drawn from the antecubital vein between 9:00 and 11:30 a.m. Samples were kept in room temperature for 1 h, centrifuged at 3500×g for 15 min at 4 °C, and frozen at −80 °C. Blood was analyzed for hormone levels at Hormone Laboratory, Oslo University Hospital, Oslo, Norway, which is accredited according to ISO 17025 standards. FSH, LH, and sex hormone-binding globulin (SHBG) were analyzed by noncompetitive immunoluminometric assays (Siemens Healthineers). Estradiol was analyzed using chemoluminescence (Diaorin Inc.), and testosterone by liquid chromatography tandem mass spectrometry (Hormone Laboratory, Oslo University Hospital, Norway). Free testosterone index (FTI) was calculated as: testosterone ×10/SHBG. The normal range for FSH, LH, estradiol, testosterone, and SHBG in adult males were 0.70–11 IU/L, 0.80–7.6 IU/L, 50–200 pmol/L, 7.2–24 nmol/L, 8–60 nmol/L, and 2.4–12, respectively. Values below the minimum detection level (DL) for FSH, LH, and estradiol were replaced with DL/√2, which has been shown to reduce left censoring bias in serum steroid measurements (Handelsman and Ly 2019). Potential presence of exogenous AAS and T/E ratio was analyzed in urine samples, as previously described (Hullstein et al. 2015). Participants who did not complete both the ERT and blood collection within a 1-week period were excluded from analyses (*n* = 15).

### Data analyses

All statistical analyses were performed using RStudio (R Core Team 2023), and assumptions for all analyses were checked using Shapiro-Wilks and Levene’s tests, along with visual checks of histograms and QQ plots. Demographic data and ERT accuracy and latency were compared among groups (*WLC/Off/On*) using ANOVA and post-hoc Tukey test to identify pairwise differences. The same analysis was repeated with the addition of age, IQ, and education as covariates. The hormone data did not meet assumptions of normality of residuals for ANOVA analysis based on Shapiro-Wilk test and visual inspection, and thus Kruskal-Wallis tests with Dunn’s all-pairs comparison test were used to compare groups. Unpaired t-tests and Mann-Whitney *U* tests were used to compare ERT accuracy and hormone measures between those with and without AAS dependence. Cohen’s *d* effect size was calculated for the group comparisons, and the effect sizes 0.2, 0.5, and 0.8 are commonly interpreted as small, medium, and large, respectively (Cohen 1988). The Benjamini-Hochberg procedure was implemented to adjust *p*-values to control for the false discovery rate (FDR) (Benjamini and Hochberg 1995), as this procedure is less susceptible to type II error than other methods of multiple testing corrections (Glickman et al. 2014). To assess possible response bias, the total number of times each emotion was selected was computed and compared between groups.

Spearman rank correlation coefficients were computed between all hormone variables to identify the most representative measure, and we selected the hormone that was most strongly correlated with the other hormone measures for subsequent analyses, which was FTI (Figure S1). To assess the relationships between FTI and ERT accuracy and response time measures, partial correlations were calculated using Spearman’s rank coefficients accounting for age. Among participants with previous or current AAS use, associations between dependence (yes/no) and ERT accuracy and latency were tested using Welch’s *t*-test and linear regression models with age, IQ, and education level as covariates. To investigate the possible mediating role of hormone levels on the relationship between AAS use and ERT performance, a mediation model was computed where FTI mediates the relationship between AAS use status and ERT measures which differed between groups and were associated with FTI. Since AAS use status was an exogenous categorical variable, indicator variables (0/1) were created, with *Off* and *On* groups entered as independent variables in the mediation analysis, and ERT accuracy as the dependent variable. In mediation models, the indirect effect is defined as the effect of the independent variable (AAS use status) on the outcome (ERT metric) through the mediator (FTI), whereas the direct effect is the effect of the independent variable on the outcome without accounting for the mediator. The total effect is calculated as the sum of the direct and indirect paths. Age, IQ, and years of education were included as covariates in the mediation model, where FTI was regressed on age, and ERT accuracy was regressed on age, IQ, and education. The mediation model was computed using structural equation modeling with the R package *lavaan* (Rosseel 2012), and confidence intervals were computed using 10,000 bootstrap samples.

All analyses were first done as complete case analysis, where all participants with complete ERT and hormone data were included. As a sensitivity analysis, missing hormone data was imputed with multiple imputation by chained equations using the R package *mice*, based on available hormone data and AAS use status (van Buuren and Groothuis-Oudshoorn 2011). Ten imputed data sets were computed using predictive mean matching, and estimates for correlation and mediation analyses were pooled using Rubin’s rules (Rubin 1996).

### Ethics

Prior to participation, all participants received a brochure with a description of the study, and written informed consent was collected. The study was conducted in accordance with the Declaration of Helsinki and received ethical approval from the Regional Committee for Medical and Health Research Ethics in South-Eastern Norway (2013/601).

## Results

### Group comparisons

The final sample consisted of 156 participants (*WLC* = 77, *Off* = 34, *On* = 45) (Table 1). The *WLC* group demonstrated higher average IQ and more years of education relative to both *On* and *Off* groups. The *On* group had higher mean weight (*p* = 0.001) and BMI (*p* = 0.002) compared to *WLC*.

**Table 1:** Demographic and AAS use characteristics of the study population

Characteristic	WLC, *N* = 77	Off, *N* = 34	On, *N* = 45	*F*	*p*
Age (mean (SD))	36.31 (8.51)	35.65 (7.95)	38.69 (10.55)	1.37	0.30
Education	16.40 (2.52)	14.55 (2.29)	14.77 (2.07)	10.48	< 0.001^a,b^
IQ	116.19 (9.11)	109.24 (10.22)	107.64 (9.61)	13.63	< 0.001^a,b^
Height	181.16 (6.35)	181.62 (6.86)	181.87 (7.10)	0.17	0.80
Weight	92.56 (11.23)	97.76 (14.81)	102.05 (16.59)	6.90	0.001^b^
BMI	35.88 (3.87)	34.35 (4.34)	33.10 (4.32)	6.69	0.002^b^
AAS initiation age	NA	20.71 (4.00)	21.91 (6.17)	0.98	0.30
Total years used	NA	8.60 (6.76)	11.36 (6.84)	3.18	0.08
Weekly dose	NA	1,010.91 (605.37)	1,012.56 (760.69)	0.00	0.99
AAS dependence (*n*/*N* (%))^†^	NA	18/34 (53%)	29/45 (64%)	0.64	0.42
Months since quitting (median (IQR))	NA	36 (24, 50)	NA		

^†^Chi-square test

^a^WLC vs Off *p* < 0.05

^b^WLC vs On *p* < 0.05

**Fig. 2:**
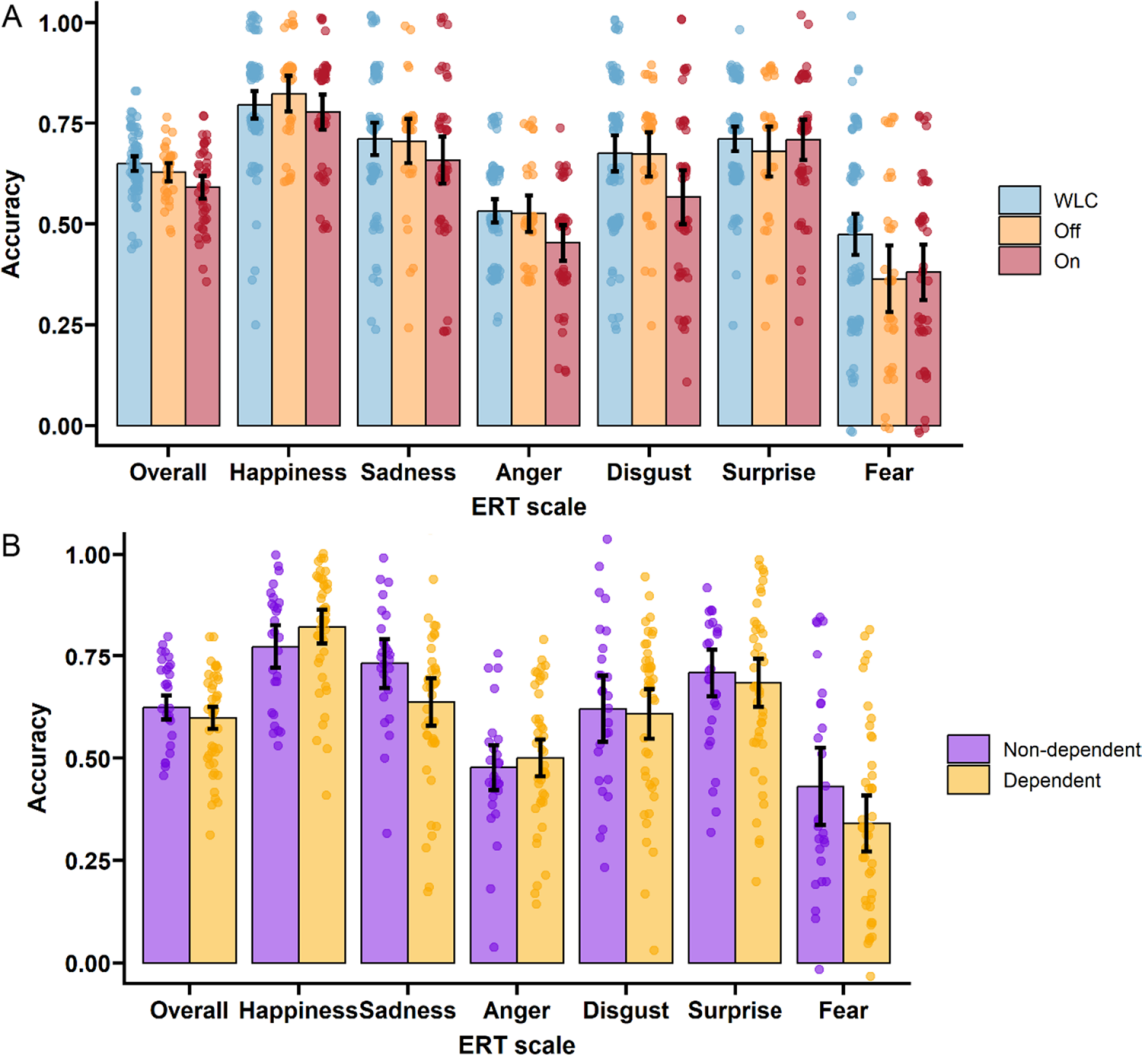
Group distributions on ERT accuracy measures. The black bar represents the group mean and 95% confidence intervals. **A** On/Off/WLC groups and **B** dependent/non-dependent groups

Results of pairwise comparisons on ERT and hormone measures can be found in Table 2, and group distributions are represented in Fig. 2. After adjusting for age, IQ, and education level, the *On* group demonstrated lower accuracy in overall emotion recognition (*Cohen’s d* = −0.68, *p*_*FDR*_ = 0.01) and recognition of anger (*d* = −0.57, *p*_*FDR*_ *=* 0.03) and disgust (*d* = −0.51, *p*_*FDR*_ = 0.05) compared to *WLC*. No pairwise group differences were identified for overall response time or for any individual emotion (Table S1). Statistically significant differences were identified between the *On* group and both *WLC* and *Off* for all measured hormones. The *Off* group demonstrated lower testosterone, SHBG, FSH, LH, and FTI and elevated T/E relative to *WLC*. Results of group comparisons of hormone levels based on imputed data can be found in the supplementary material (Table S2). No statistically significant differences in number of responses per emotion were identified between the groups (Table S3, Figure S2).

**Table 2:** Comparison of ERT accuracy and overall latency and hormones among *On* AAS, *Off* AAS, and WLC groups. CANTAB ERT tests statistics and significance values are based on Tukey’s HSD test, hormone comparisons are based on Kruskal-Wallis

Measure	WLC, *N* = 77^1^	Off, *N* = 34^1^	On, *N* = 45^1^	F/H^2^	*d* WLC vs. Off	*d* WLC vs. On	d Off vs. On	*F*^3^
Overall RT	1592.19 (628.31)	1653.90 (628.63)	1560.73 (511.24)	0.24	0.10	−0.06	−0.16	0.12
Overall	0.65 (0.08)	0.63 (0.07)	0.59 (0.09)	7.48**	−0.29	−0.68**	−0.47	6.87*^†^
Happiness	0.80 (0.15)	0.82 (0.13)	0.78 (0.15)	0.99	0.20	−0.12	−0.33	0.76
Sadness	0.71 (0.18)	0.71 (0.16)	0.66 (0.19)	1.34	−0.03	−0.28	−0.27	1.17
Anger	0.53 (0.13)	0.53 (0.13)	0.45 (0.15)	5.31*	−0.05	−0.57*	−0.57	4.93*^†^
Disgust	0.68 (0.20)	0.67 (0.16)	0.57 (0.22)	4.76*	0.01	−0.51*	−0.55	4.53*^†^
Surprise	0.71 (0.14)	0.68 (0.18)	0.71 (0.17)	0.50	−0.2	−0.02	0.16	0.64
Fear	0.47 (0.23)	0.36 (0.24)	0.38 (0.23)	3.80*	−0.48	−0.41	0.07	3.21
Testosterone	19.74 (7.13)	13.49 (7.58)	44.06 (41.79)	20.22***	−0.85**	0.81*	1.02***	
Missing	31	6	15					
TE	1.19 (1.02)	1.83 (1.78)	54.97 (49.24)	75.16***	0.44	1.54***	1.53***	
Missing	6	2	5					
SHBG	42.91 (19.89)	32.57 (16.26)	19.61 (15.77)	30.26***	−0.57*	−1.30***	−0.81**	
Missing	30	4	14					
FSH	5.25 (3.02)	3.78 (2.38)	0.67 (2.01)	55.17***	−0.54*	−1.79***	−1.41***	
Missing	30	4	13					
LH	4.76 (1.81)	3.50 (1.66)	0.25 (0.71)	67.26***	−0.73*	−3.27***	−2.54***	
Missing	30	4	13					
Estradiol	82.00 (31.17)	78.41 (28.05)	204.60 (184.05)	15.15***	−0.12	0.93**	0.96**	
Missing	29	5	15					
FTI	4.87 (1.30)	4.24 (2.41)	65.73 (121.72)	23.94***	−0.32*	0.71***	0.71***	
Missing	31	5	15					

*RT* reaction time, *TE* testosterone/epitestosterone ratio, *SHBG* sex hormone-binding globulin, *FSH* follicle-stimulating hormone, *LH* luteinizing hormone, *FTI* free testosterone index, *d* Cohen’s *d*

***p < 0.001, **p < 0.01, *p < 0.05 (false discovery rate adjusted)

^1^Mean (SD)

^2^*F* statistic for ERT measures, *H* statistic for hormones

^3^*F* statistic adjusted for age, IQ, and education

^†^pFDR < 0.05 WLC vs On based on post-hoc Tukey HSD

### Dependence

Among participants reporting lifetime AAS use, 47 met criteria for AAS dependence. Those with AAS dependence indicated lower accuracy in recognition of sadness (*d* = 0.53, *p* = 0.02, *p*_*FDR*_ = 0.13) and fear (*d* = 0.51, *p* = 0.03, *p*_*FDR*_ = 0.13), relative to those without AAS dependence (*n* = 32). In addition, those with AAS dependence had lower levels of SHBG (*p* = 0.03, *p*_*FDR*_ = 0.08), FSH (*p* = 0.01, *p*_*FDR*_ = 0.08), and LH (*p* = 0.03, *p*_*FDR*_ = 0.08) relative to those without dependence (Table 3, imputed data Table S4). After linear regression adjusting for age, IQ, and education, only fear was associated with AAS dependence prior to FDR adjustment (*d* = 0.58, *p* = 0.03, *p*_*FDR*_ = 0.24) (Table 4).

**Table 3:** Mean (SD) and results of unpaired *t*-test of ERT accuracy and overall latency, and Mann-Whitney *U* test of hormones among dependent and non-dependent AAS consumers

Characteristic	Non-dependent, *N* = 32	Dependent, *N* = 47	*t*/*U*^1^	*d*	*p*	*p* FDR
Overall RT	1640.38 (529.32)	1573.90 (588.65)	0.52	0.12	0.60	0.80
Overall	0.63 (0.07)	0.59 (0.09)	1.80	0.40	0.08	0.20
Happiness	0.78 (0.14)	0.81 (0.14)	−1.07	−0.24	0.29	0.58
Sadness*	0.73 (0.15)	0.64 (0.19)	2.42	0.53	0.02	0.13
Anger	0.48 (0.13)	0.48 (0.15)	0.01	0.00	0.99	0.99
Disgust	0.61 (0.20)	0.61 (0.21)	−0.11	−0.02	0.92	0.99
Surprise	0.71 (0.14)	0.68 (0.19)	0.85	0.18	0.40	0.64
Fear*	0.44 (0.23)	0.33 (0.22)	2.19	0.51	0.03	0.13
Testosterone	22.97 (17.79)	33.77 (41.49)	420	−0.32	0.85	0.85
*Missing*	*8*	*13*				
TE	18.11 (31.88)	40.81 (50.94)	466	−0.52	0.06	0.11
*Missing*	*2*	*5*				
SHBG*	33.12 (20.51)	21.03 (12.45)	599	0.75	0.03	0.08
*Missing*	*7*	*11*				
FSH*	3.18 (3.15)	1.49 (2.10)	630	0.66	0.01	0.08
*Missing*	*7*	*10*				
LH*	2.54 (2.21)	1.34 (1.82)	600	0.60	0.03	0.08
*Missing*	*7*	*10*				
Estradiol	124.67 (112.11)	154.86 (166.02)	448	−0.21	0.66	0.82
*Missing*	*8*	*12*				
FTI	12.46 (18.28)	51.31 (115.86)	394	−0.43	0.70	0.82
*Missing*	*8*	*12*				

*d* Cohen’s *d*, *FDR* false discovery rate, *RT* reaction time

***p < 0.001, **p < 0.01, *p < 0.05

^1^*T* statistic for ERT measures, Mann-Whitney *U* for hormones

**Table 4:** Adjusted linear regression (age, IQ, education), AAS dependence as predictor, non-dependent AAS consumers as reference group, and ERT accuracy and overall latency measures as outcome variables

Variable	*β* (95% CI)	*d*	*p*	*p* FDR	*R*2
Overall	−0.02 (−0.06 to 0.02)	0.27	0.30	0.48	0.10
Overall RT	−192.2 (−458.31 to 73.9)	0.37	0.15	0.36	0.11
Anger	0.01 (−0.07 to 0.08)	−0.06	0.81	0.81	−0.04
Disgust	0.04 (−0.06 to 0.14)	−0.22	0.39	0.52	0.04
Fear	−0.13 (−0.25 to −0.02)	0.58	0.03	0.24	0.02
Happiness	0.05 (−0.02 to 0.12)	−0.35	0.18	0.36	−0.01
Sadness	−0.08 (−0.16 to 0.01)	0.44	0.09	0.36	0.09
Surprise	−0.02 (−0.10 to 0.07)	0.11	0.66	0.75	0.06

*FDR* false discovery rate, *d* Cohen’s *d*, *RT* reaction time

### FTI and ERT correlation

**Fig. 3:**
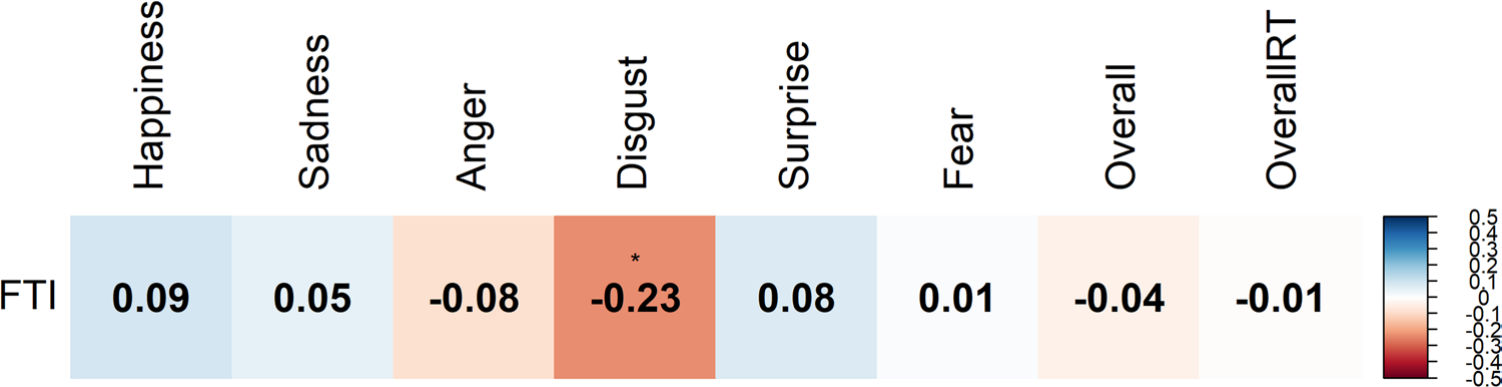
Correlation among ERT accuracy and overall latency measures and free testosterone index, correlations with Spearman’s rho, adjusted for age. ***p < 0.001, **p < 0.01, *p < 0.05

Results of correlation analyses can be found in Fig. 3. Recognition of disgust was negatively correlated with FTI (*ρ* = −0.23, *p* = 0.02). Results of correlation analyses on imputed data can be found in supplementary materials (Table S5).

### Mediation analysis

**Fig. 4:**
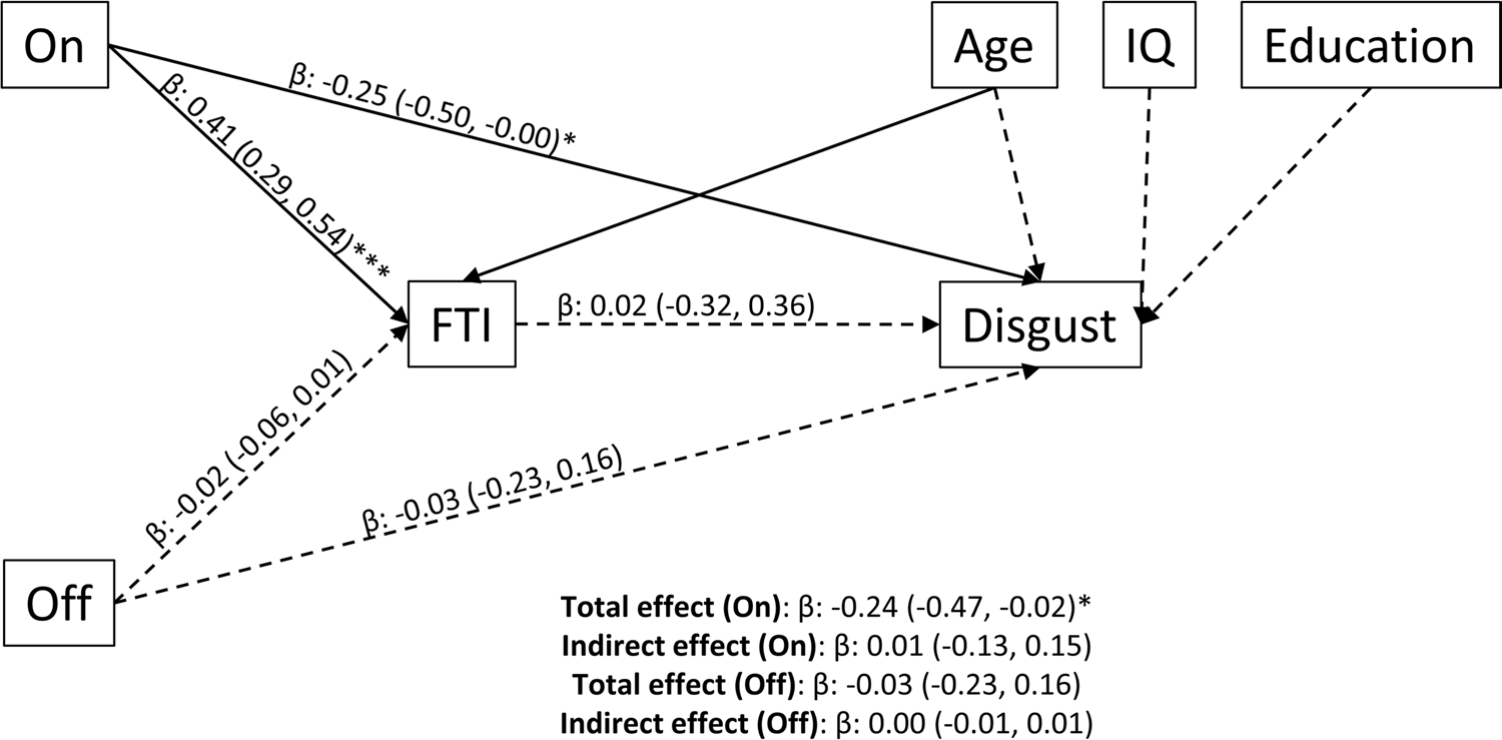
Mediation model with FTI as the mediator between AAS use status and accuracy of emotional recognition of disgust. Results presented as standardized regression coefficients with 95% confidence intervals. Dashed lines represent non-significant (*p* > 0.05) paths. β, standardized coefficients; FTI, free testosterone index. ***p < 0.001, **p < 0.01, *p < 0.001

FTI was investigated as a mediator of the association between AAS use and recognition of disgust. Fig. 4 depicts the standardized coefficients and 95% confidence intervals of the paths. Relative to *WLC* and *Off*, current AAS use (*On*) was associated with an increase in FTI (*β* = 0.41, *p* = 0.01). Current AAS use was associated with poorer recognition of disgust (*β* = −0.27, *p* = 0.04), and the total effect of current AAS use (*On*) on disgust was statistically significant; however, the indirect effect (pathway from *On* to disgust through FTI) was not statistically significant. Full results including non-standardized coefficients and fit statistics in addition to results of the mediation models on imputed data can be found in the supplementary materials (Tables S6-S7).

## Discussion

This study demonstrated poorer facial emotion recognition among males currently using anabolic steroids (*On*) (AAS) relative to weight-lifting controls (WLC), primarily when the task involved facial expressions depicting anger and disgust. Furthermore, while associations with FTI levels and recognition of disgust were identified, we did not find a significant mediating effect of FTI levels on the relationship between AAS use and emotion recognition.

In line with our hypothesis, we identified group differences suggesting that current AAS use is associated with impaired emotional recognition, mainly driven by poorer accuracy in recognition of disgust and anger. These findings align with previous studies from our research group that demonstrate impaired theory of mind and emotion recognition of biological movement in a sample partially overlapping with the current study sample (Hauger et al. 2019a; Vaskinn et al. 2020). This suggests that AAS use is associated with impaired performance on various social cognitive abilities. Notably, males who had previously used AAS (*Off* group) did not significantly differ from controls, consistent with the interpretation that AAS use may temporarily impact the ability to identify emotions from facial expressions. The on-cycle group exhibited a distinct hormonal profile compared to both WLC and off-cycle users, including lower levels of LH, FSH, and SHBG, and elevated serum testosterone level and E2 levels. Given previous studies indicating hormonal influence on emotion recognition (Kiyar et al. 2022), hormonal shifts during on-cycle use identified in this study support a relationship between hormonal changes and facial emotion recognition. Previous findings suggest that other characteristics of AAS use, primarily age of initiation and duration of use may impact cognition (Bjørnebekk et al. 2019; Hildebrandt et al. 2014; Kanayama et al. 2013). We did not identify any statistically significant differences in age of initiation or accumulated years of use between the *On/Off* groups in the current study, therefore these characteristics are unlikely to explain the group differences. It is possible that our findings can be partially explained by psychiatric variables not taken into account but associated with both AAS use and social cognitive deficits, such as personality disorders and attention-deficit/hyperactivity disorder symptoms (Daros et al. 2013; Kildal et al. 2022; Marissen et al. 2012; Morellini et al. 2022). Alternatively, male athletes have reported feeling invincible and superior to others while taking AAS, which may be a cause or consequence of decreased empathic behavior (Vassallo and Olrich 2010).

We identified putative associations between AAS dependence and decreased recognition of sadness and fear; however, these results did not remain significant after FDR correction and/or adjustment for age, IQ, and education. Thus, our hypothesis that men with AAS dependence would demonstrate poorer emotion recognition than men without dependence was not supported by the current findings. Previous research from our group in a partially overlapping sample has identified associations between AAS dependence and impaired recognition from biological motion of fear, suggesting that the unadjusted association in the current study may be meaningful, as there may be small differences in social cognition that the current study lacked power to identify (Hauger et al. 2019a). In addition, while IQ has been associated with social cognition, traditional and emotional intelligence are two distinct cognitive domains, and “controlling” for group differences in this case may not be appropriate (Lawrence et al. 2015; Miller and Chapman 2001; Mohn et al. 2014; Roberts et al. 2001). Putative associations between AAS dependence and recognition of fear and sadness are important as difficulties recognizing these emotions may have implications for interpersonal relationships. Moreover, similar deficits have been associated with paranoid and antisocial beliefs, and history of violence (Bulgari et al. 2020; Hanegraaf et al. 2020).

Interestingly, higher levels of FTI and testosterone were associated with increased overall response time, indicating slower speed of processing of facial stimuli. While some studies indicate that endogenous testosterone is positively associated with cognitive performance, and appears protective of cognitive functioning, particularly in older men, supraphysiological doses may impair cognitive functioning (Hauger et al. 2020; Hildebrandt et al. 2014; Hua et al. 2016). Based on these previous findings, and considering that our sample is relatively young, we hypothesized that androgen levels would partially mediate the relationship between AAS use and ERT performance. In contrast to our hypothesis, the mediation model provided no evidence to support that FTI levels mediated the observed association between AAS use status and recognition of disgust. In our study, we found that FTI was the best measure for distinguishing between active users and non-users. This could be attributed to the fact that FTI reflects the free fraction of testosterone. Active use of AAS may decrease or increase total testosterone, depending on the particular substances used, because of the influence of testosterone on SHBG. FTI serves as a correction to this issue. Additionally, the T/E ratio represents an alternative to testosterone measurements. It is important to acknowledge the strong correlations among the measured hormones, posing a challenge in discerning their specific impact on ERT performance. Exogenous testosterone leads to increased estradiol due to its conversion via the enzyme CYP19A1 (Schiffer et al. 2019). This conversion may attenuate the effect of testosterone on the brain over time, and it has been suggested that some behavioral effects typically attributed to testosterone might be driven by estradiol (Gamsakhurdashvili et al. 2021; Mueller et al. 2011). This dynamic could also apply to facial emotion recognition. Additionally, the absence of a significant mediating effect of FTI on the relationship between AAS use and emotion recognition might result from using a single hormone measure, when the emotion recognition abilities of men currently on-cycle are likely influenced by several hormones which may have divergent effects.

The lack of a significant mediation effect by FTI may be explained by unmeasured factors associated with both AAS use and ERT. Previous studies testing the association between testosterone and social cognition have reported both positive and negative associations (Lausen et al. 2020; Nadler et al. 2019; Vongas and Al Hajj 2017). This heterogeneity in results is potentially a result of complex interactions between environmental and biological variables. For example, relationships between testosterone and social cognition and behaviors can be moderated by cortisol or competition (Carré et al. 2014; Lausen et al. 2020; Wagels et al. 2018).

Further, single blood-based measures of hormones cannot fully capture endocrine disturbances resulting from current or previous AAS use. Additional factors including androgen receptor density and other biomarkers indicating the functioning of neuroendocrine systems including the hypothalamic-pituitary-gonadal axis as well as oxytocin and vasopressin may provide better understanding of the relationship between AAS use and social cognition (Karlsson et al. 2016; Lu et al. 2019; Zink and Meyer-Lindenberg 2012).

AAS use has been shown to influence several complex neuropsychiatric traits, and thus the current findings may be explained by additional factors which are more proximal to social cognitive behaviors, including structural and functional changes to relevant brain regions including the prefrontal cortex and amygdala (Hauger et al. 2019b; Hiser and Koenigs 2018; Kaufman et al. 2015; Westlye et al. 2017). Testosterone has been shown to decrease connectivity between the right amygdala and dorsolateral prefrontal cortex, and increase neural reactivity to fearful and angry expressions in the amygdala, with implications for emotional recognition and behavioral response (Derntl et al. 2009; Goetz et al. 2014; Votinov et al. 2020). In addition, a previous study of transgender men demonstrated changes in emotional processing following gender-affirming hormone therapy, where neural patterns shifted from those consistent with sex assigned at birth to those consistent with gender identity 6–10 months following treatment initiation (Kiyar et al. 2022). While the current study cannot conclude a causal effect of AAS on emotion recognition, these findings suggest that exogenous testosterone leads to functional changes in brain areas required for emotional processing including the amygdala and ACC. Previous findings from our research group (in a partially overlapping sample at an earlier time point) indicate decreased connectivity between the default mode network and amygdala, and between the dorsal attention network and regions implicated in attentional control, impulse inhibition, and executive functions among men using AAS, with further reductions among men who were currently on-cycle or with AAS dependence (Westlye et al. 2017). Decreased connectivity in these regions likely impact emotional processing and performance on the ERT task. Additionally, the further reductions in connectivity demonstrated in on-cycle users may partially explain the difference between the *On* and *Off* groups recognition of disgust and anger seen in the current study.

### Limitations

Several limitations of the current study should be noted. ERT performance may be influenced by mental state, motivation and mood. For example, it may be more difficult to recognize emotions that are incongruent with one’s own emotions (Schmid and Mast 2010). To combat this, we facilitated standardized test situations in a controlled environment; however, structured test situations may not reflect emotion recognition ability in everyday environments requiring complex simultaneous processing of signals from face, voice, and body movements. ERT is regarded as a valuable tool for measuring emotional perception (Montagne et al. 2007) and appears to produce stable findings over time (Gica et al. 2019), yet the ecological validity is limited due to the limited range of emotions and singular focus (facial expressions), which is not representative of a real world setting (Schlegel et al. 2014). Using a more ecologically valid video task, we previously found impaired theory of mind performance in AAS (Vaskinn et al. 2020). The six emotions addressed in this study are regarded to have universal signal value (Ekman and Friesen 1971), though some research indicates that emotion recognition is culturally dependent (Mohan et al. 2021; Prado et al. 2014). Additionally, we used a shortened version of the ERT, which may lack the experimental power to identify subtle differences in emotion recognition processing.

While data included in the hormone and ERT correlations analyses were restricted to those who completed both within the same week, the exact AAS regiment of current users was not factored into the analysis (i.e., if they took AAS that day, the previous day). Furthermore, the exclusion of individuals who had a larger gap between tests (*n* = 15) may contribute to selection bias. There is also a significant portion of missing hormone data within the WLC group due to practical challenges in data collection, which may bias our findings although all analyses including hormone data were repeated after multiple imputation for these variables. Additional unmeasured variables may confound the relationship between current AAS use and facial emotion recognition. For example, polysubstance use is common among people using AAS, and may confound the relationship between AAS use and antisocial behaviors, as polysubstance use is associated with impaired emotion recognition (Fernández-Serrano et al. 2010; Lundholm et al. 2015; Sagoe et al. 2015). In addition, the current operationalization of AAS dependence is a lifetime measurement, and may not accurately reflect the dependence status at the time of ERT administration. The findings are also based on cross-sectional data and cannot make any claims about causality. Since this is an all-Norwegian all-male sample, the generalizability to other contexts and to females is unclear. Finally, the group sizes may not be sufficiently powered to identify associations with small effect sizes. Thus, the results of these analyses should be viewed as exploratory and interpreted with caution.

## Conclusion

The present study identified putative deficits in facial emotional recognition among males currently using AAS use relative to controls. However, this association did not appear to be mediated by serum testosterone levels, suggesting other mechanisms should be explored. Future research is required to determine possible effects of high doses of exogenous androgens on oxytocin, vasopressin, and other neuroendocrine variables involved in social cognition and behavior. In addition, future studies should further explore potential differences between people with and without AAS dependence using a task which may be able to identify more subtle differences in processing, such as a face morphing task. The current findings also indicate a potential temporal effect of AAS use, as males who ceased use did not demonstrate any significant differences in emotion recognition compared to controls, despite previous findings indicating long-term effects on neuroendocrine systems. Additional longitudinal research is required to determine the long-term and dynamic effects of AAS use on social cognition, and subsequent effects on antisocial or aggressive behavior.

### Supplementary information

ESM 1 (DOCX 586 kb)
