# Supplementary material for: Supraphysiological testosterone levels from anabolic steroid use and reduced sensitivity to negative facial expressions in men
Source: Psychopharmacology (Berl). 2023 Nov 22;241(4):701–15. doi: 10.1007/s00213-023-06497-2 (PMC10927904; doi:10.1007/s00213-023-06497-2)
Supplement: Supplementary file 1 — (DOCX 586 kb) [file 213_2023_6497_MOESM1_ESM.docx]

Supplementary materials

**Figure S1**. Spearman’s correlation coefficients of all measured hormones.


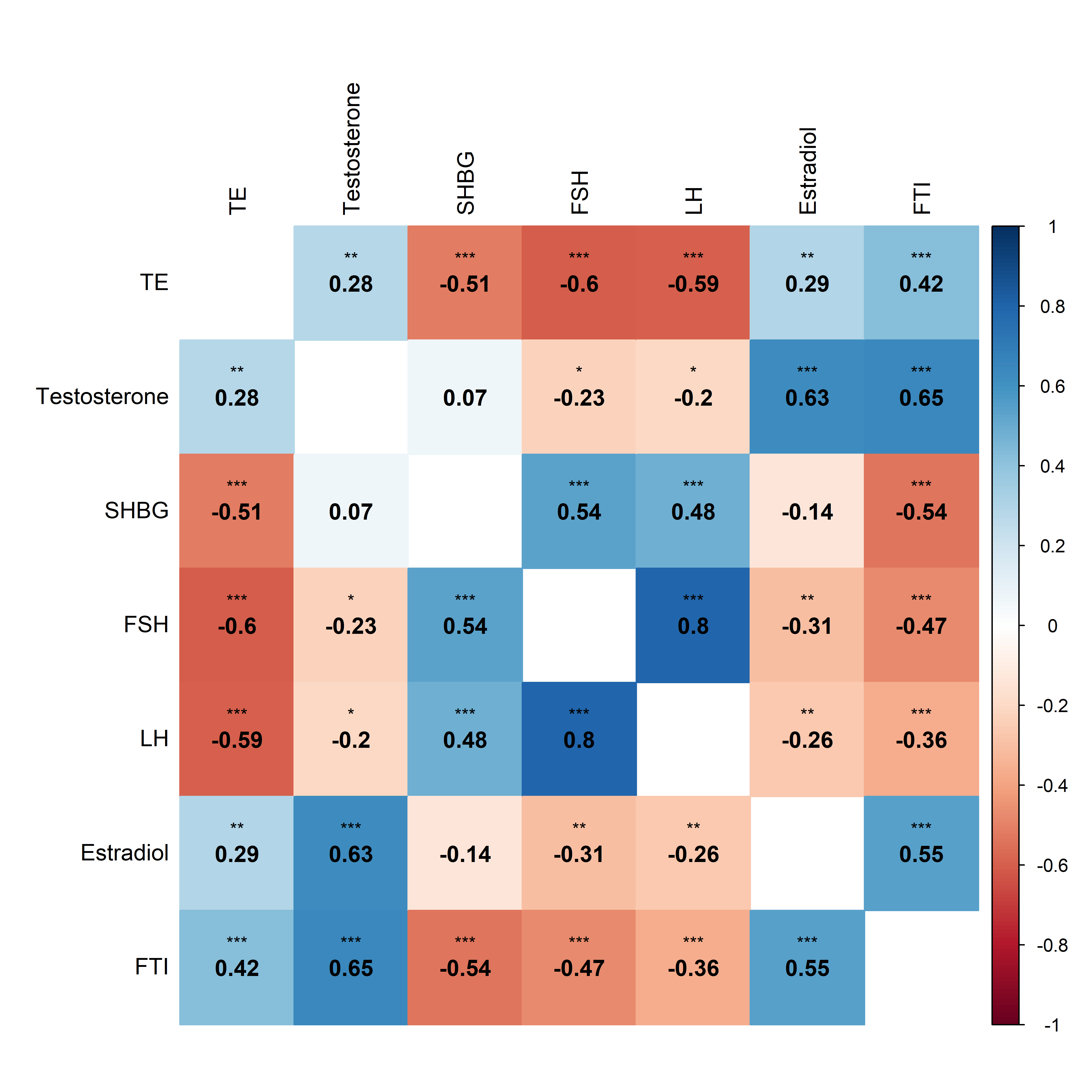


| **Table S1.** Comparison of ERT reaction time among On AAS, Off AAS and WLC groups, F statistic and statistical significance from one-way ANOVA, Cohen’s d calculated for pairwise group comparisons. | | | | | | | |
| --- | --- | --- | --- | --- | --- | --- | --- |
| **Measure** | **WLC**, N = 77*^1^* | **Off**, N = 34 | **On**, N = 45 | **F** | **d WLC vs. Off** | **d WLC vs. On** | **d Off vs. On** |
| Overall | 1,592.19 (628.31) | 1,653.90 (628.63) | 1,560.73 (511.24) | 0.24 | 0.10 | -0.06 | -0.16 |
| Anger | 1,502.88 (1,146.21) | 1,477.31 (874.41) | 1,380.11 (562.90) | 0.24 | -0.03 | -0.14 | -0.13 |
| Disgust | 2,216.95 (1,083.87) | 2,029.15 (901.97) | 2,230.16 (1,164.19) | 0.43 | -0.19 | 0.01 | 0.19 |
| Fear | 2,345.54 (1,109.79) | 2,746.88 (1,545.45) | 2,464.79 (1,175.01) | 1.20 | 0.30 | 0.10 | -0.21 |
| Happiness | 1,118.51 (603.34) | 1,209.79 (535.63) | 1,159.62 (448.06) | 0.34 | 0.16 | 0.08 | -0.10 |
| Sadness | 1,826.73 (668.33) | 2,198.87 (954.10) | 1,832.30 (883.23) | 2.83 | 0.45 | 0.01 | -0.40 |
| Surprise | 1,324.88 (759.43) | 1,687.22 (1,257.47) | 1,343.03 (624.35) | 2.28 | 0.35 | 0.03 | -0.35 |

| **Characteristic** | **H WLC vs. Off** | **H WLC vs. On** | **H Off vs. On** | **p WLC vs. Off** | **p WLC vs. On** | **p Off vs. On** |
| --- | --- | --- | --- | --- | --- | --- |
| Testosterone | 4.0 | 3.0 | 6.1 | <0.001 | <0.001 | <0.001 |
| TE | 1.6 | 9.1 | 6.0 | 0.034 | <0.001 | <0.001 |
| SHBG | 3.3 | 6.2 | 2.1 | <0.001 | <0.001 | 0.007 |
| FSH | 2.6 | 9.3 | 5.3 | <0.001 | <0.001 | <0.001 |
| LH | 2.7 | 10 | 5.8 | <0.001 | <0.001 | <0.001 |
| Estradiol | 0.44 | 4.9 | 4.4 | 0.7 | <0.001 | <0.001 |
| FTI | 2.8 | 3.9 | 5.7 | <0.001 | <0.001 | <0.001 |

**Table S2**. Results of Kruskal-Wallis with Dunn’s test pairwise comparisons of hormones based on multiple imputation

*TE: testosterone/epitestosterone ratio, SHBG: sex hormone-binding globulin, FSH: follicle-stimulating hormone, LH: luteinizing hormone, FTI: free testosterone index*

| **Table S3.** Mean and (SD) of total number of responses for each emotion by group, p-values are adjusted to control for false discovery rate. | | | | | | |
| --- | --- | --- | --- | --- | --- | --- |
| **Characteristic** | **WLC**, N = 77 | **Off**, N = 34 | **On**, N = 45 | **F WLC vs. Off** | **F WLC vs. On** | **F Off vs. On** |
| Happiness | 8.13 (3.00) | 9.44 (3.97) | 7.78 (2.09) | 2.98 | 0.88 | 3.43 |
| Sadness | 8.99 (3.17) | 9.03 (3.37) | 9.11 (3.66) | 0.09 | 0.28 | 0.15 |
| Anger | 5.68 (1.73) | 5.35 (1.84) | 5.49 (2.36) | 1.13 | 0.72 | 0.43 |
| Disgust | 9.57 (3.35) | 9.21 (2.94) | 9.31 (3.94) | 0.73 | 0.57 | 0.19 |
| Surprise | 9.03 (3.06) | 9.85 (3.53) | 10.38 (4.08) | 1.63 | 2.92 | 0.94 |
| Fear | 6.61 (2.86) | 5.12 (3.32) | 5.93 (2.90) | 3.45 | 1.71 | 1.71 |

**
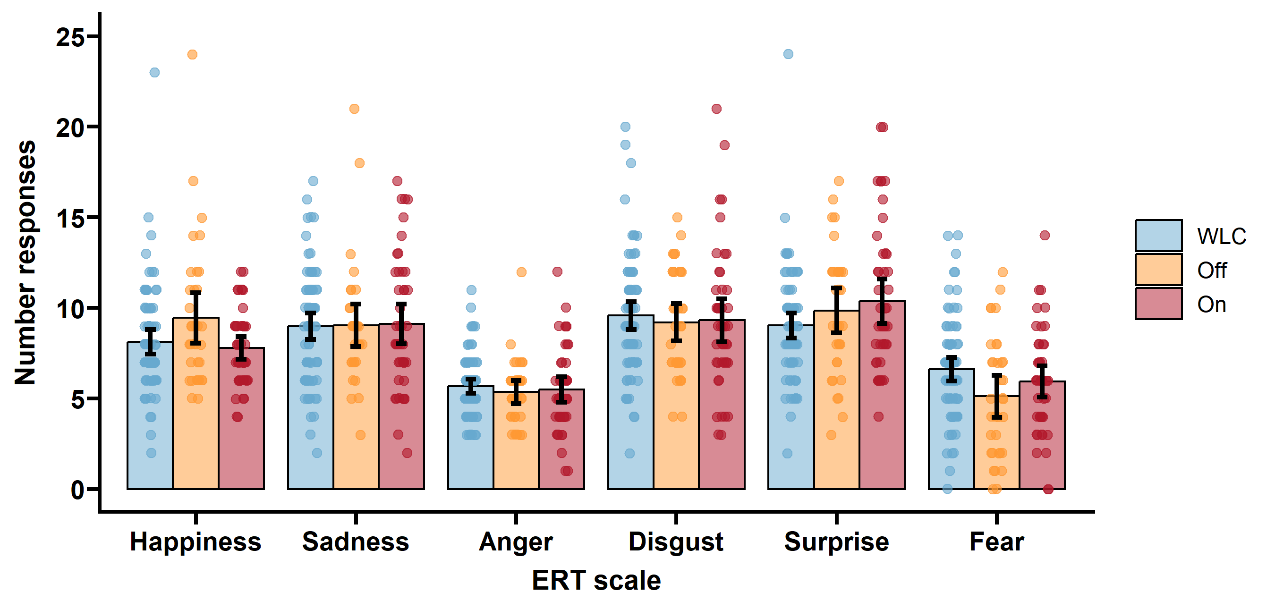
Figure S2**. Group distribution of total number of responses per emotion

**Table S4.** Results of Mann-Whitney U-test between dependent and non-dependent AAS based on multiply imputed data

| **Characteristic** | *p* | U |
| --- | --- | --- |
| Testosterone | 0.114 | 713 |
| TE | **0.013** | 547 |
| SHBG | **0.006** | 996 |
| FSH | **0.013** | 1,041 |
| LH | **0.036** | 942 |
| Estradiol | 0.181 | 714 |
| FTI | **0.032** | 620 |

*TE: testosterone/epitestosterone ratio, SHBG: sex hormone-binding globulin, FSH: follicle-stimulating hormone, LH: luteinizing hormone, FTI: free testosterone index*

**Table S5.** Partial correlation of FTI and ERT metrics based on multiply imputed data, spearman’s correlation coefficient (rho), adjusted for age.

| FTI | ρ | *p* |
| --- | --- | --- |
| OverallRT | -0.04 | 0.66 |
| Happiness | 0.07 | 0.45 |
| Sadness | 0.08 | 0.46 |
| Anger | -0.01 | 0.92 |
| Disgust | -0.05 | 0.61 |
| Surprise | -0.05 | 0.58 |
| Fear | -0.01 | 0.90 |
| Overall | -0.07 | 0.49 |

RT: reaction time, ρ = Spearman’s correlation coefficient

**Table S6.** Full results of mediation model FTI based on complete case analysis

| **Path** | **Estimate** | **95% CI** | **Standardized β** |
| --- | --- | --- | --- |
| a1: Off🡪FTI | -0.05 | -0.48, 0.37 | -0.02 |
| a2: On🡪FTI | 0.90*** | 0.48, 1.33 | 0.41 |
| b: FTI-🡪Disgust | 0.00 | -0.03, 0.04 | -0.02 |
| c1: Off🡪Disgust | -0.02 | -0.10, 0.07 | -0.03 |
| c2: On🡪Disgust | -0.12 | -0.21, 0.02 | -0.27 |
| Indirect effect Off🡪FTI🡪Disgust (a1*b) | 0.00 | -0.00, 0.00 | 0 |
| Indirect effect On🡪FTI🡪Disgust (a2*b) | 0.00 | -0.03, 0.04 | 0.01 |
| Total effect Off ((a1*b)+c1) | -0.02 | -0.10, 0.07 | -0.03 |
| Total effect On ((a1*b)+c1) | -0.11* | -0.20, -0.03 | -0.26 |
| CFI: 0.90 |  |  |  |
| RMSEA: 0.13 |  |  |  |
| SRMR: 0.04 |  |  |  |

*CFI: Comparative fit index, RMSEA: Root mean square error of approximation, SRMR: standardized root mean square residual, FTI: Free testosterone index*

**Table S7.** Results of mediation model of FTI based on multiply imputed data

| **Path** | **Estimate** | **95% CI** | **Standardized β** |
| --- | --- | --- | --- |
| a1: Off🡪FTI | -0.06 | -0.46, 0.34 | -0.02 |
| a2: On🡪FTI | 0.86*** | 0.47, 1.25 | 0.37 |
| b: FTI-🡪Disgust | 0.00 | -0.03, 0.04 | -0.02 |
| c1: Off🡪Disgust | 0.04 | -0.05, 0.12 | 0.08 |
| c2: On🡪Disgust | -0.09 | -0.17, 0.00 | -0.19 |
| Indirect effect Off🡪FTI🡪Disgust (a1*b) | 0.00 | -0.00, 0.00 | 0.00 |
| Indirect effect On🡪FTI🡪Disgust (a2*b) | 0.00 | -0.03, 0.03 | 0.01 |
| Total effect Off ((a1*b)+c1) | 0.04 | -0.05, 0.12 | 0.07 |
| Total effect On ((a1*b)+c1) | -0.08* | -0.17, -0.00 | -0.18 |
| CFI: 0.90 |  |  |  |
| RMSEA: 0.11 |  |  |  |
| SRMR: 0.04 |  |  |  |

*CFI: Comparative fit index, RMSEA: Root mean square error of approximation, SRMR: standardized root mean square residual, FTI: Free testosterone index*
